# Targeting of mutant *hogg1 *in mammalian mitochondria and nucleus: effect on cellular survival upon oxidative stress

**DOI:** 10.1186/1471-2407-6-235

**Published:** 2006-10-03

**Authors:** Aditi Chatterjee, Elizabeth Mambo, Yonggang Zhang, Theodore DeWeese, David Sidransky

**Affiliations:** 1 Department of Otolaryngology-Head and Neck Surgery, Head and Neck Cancer Research Division, 1550 Orleans Street, Johns Hopkins University School of Medicine, Cancer Research Building II, Room 5 06, Baltimore, MD 21231, USA; 2 Department of Radiation Oncology and Molecular Radiation Sciences, Johns Hopkins University, Baltimore, MD 21231, USA

## Abstract

**Background:**

Oxidative damage to mitochondrial DNA has been implicated as a causative factor in a wide variety of degenerative diseases, aging and cancer. The modified guanine, 7,8-dihydro-8-oxoguanine (also known as 8-hydroxyguanine) is one of the major oxidized bases generated in DNA by reactive oxygen species and has gained most of the attention in recent years as a marker of oxidative DNA injury and its suspected role in the initiation of carcinogenesis. 8-hydroxyguanine is removed by hOgg1, a DNA glycosylase/AP lyase involved in the base excision repair pathway.

**Methods:**

We over-expressed wild type and R229Q mutant *hOGG1 *in the nucleus and mitochondria of cells lacking mitochondrial *hOGG1 *expression through an expression vector containing nuclear and mitochondrial targeting sequence respectively. We used quantitative real time PCR to analyze mtDNA integrity after exposure to oxidative damaging agents, in cells transfected with or without mitochondrially-targeted mutant *hogg1*.

**Result:**

Over-expression of wild type hOgg1 in both nucleus and mitochondria resulted in increased cellular survival when compared to vector or mutant over-expression of hOGG1. Interestingly, mitochondrially-targeted mutant hogg1 resulted in more cell death than nuclear targeted mutant hogg1 upon exposure of cells to oxidative damage. Additional we examined mitochondrial DNA integrity after oxidative damage exposure using real-time quantitative PCR. The presence of mutant *hogg1 *in the mitochondria resulted in reduced mitochondrial DNA integrity when compared to the wild type. Our work indicates that the R229Q *hOGG1 *mutation failed to protect cells from oxidative damage and that such mutations in cancer may be more detrimental to cellular survival when present in the mitochondria than in the nucleus.

**Conclusion:**

These findings suggest that deficiencies in *hOGG1*, especially in the mitochondria may lead to reduced mitochondrial DNA integrity, consequently resulting in decreased cell viability.

## Background

The detection of mitochondrial DNA (mtDNA) mutations in several human diseases has stimulated interest in understanding how the integrity of the mitochondrial genome is maintained [1-4]. It is believed that these mutations likely result from the exposure of mtDNA to reactive oxygen species (ROS). mtDNA is continuously exposed to ROS which are formed as byproducts of normal cell metabolism and during exposure to physical and chemical agents such as γ-irradiation, UV-irradiation or H_2_O_2. _Lack of protective histones, proximity to oxidative phosphorylation and limited capacity for repair of DNA damage [5-7] predispose mtDNA to attack by ROS. ROS such as hydroxyl radical (OH^•^), superoxide radical (O_2_^-^), and singlet oxygen (^1^O_2_), damage DNA directly [8], inducing a wide range of DNA lesions that include single and double strand DNA breaks, apurinic apyrimidinic (AP) sites, DNA-protein-cross-links and oxidized DNA bases [8,9]. Among the oxidized bases, the modified guanine, 7,8-dihydro-8-oxoguanine (8-oxoG) (also known as 8-hydroxyguanine) is one of the major lesions generated in DNA by oxygen radicals [9] and has gained most of the attention in recent years as a marker of oxidative DNA injury and its suspected role in the initiation of carcinogenesis [10,11]. It has been shown that the presence of 8-oxoG results in incorporation of deoxyadenosine triphosphate (dATP) opposite 8-oxoG during replication yielding G : C to T : A transversions [10,12-14]. Since 8-oxoG constitutes a premutagenic lesion, efficient repair mechanisms are vital to prevent these lesions from becoming permanent mutations.

To prevent the deleterious action of 8-oxoG, living organisms have evolved specific DNA repair mechanisms for this biologically important lesion. *E. coli*. possess the GO system, [15] which consists of three genes namely: MutT, MutM and MutY. MutT, encodes a phosphatase that hydrolyzes 8-oxoGTP in the nucleotide pool to 8-oxoGMP, thus preventing incorporation of 8-oxoGTP during DNA replication, while MutM is a DNA glycosylase/AP lyase that preferentially removes 8-oxoG opposite cytosine. MutY is a DNA glycosylase that specifically removes adenine opposite 8-oxoG. The existence of these three genes in *E. coli *for the repair of 8-oxoG supports the fundamental biological importance of this lesion. A MutM homologue, called *OGG1*, was isolated from *Saccharomyces serevisiae *in 1996 [16,17]. Several groups independently isolated a human homologue of *OGG1 *(*hOGG1*) [18-21]. The *hOGG1 *gene encodes two major isoforms, α-*hOGG1 *and β-*hOGG1 *that are products of alternative splicing. α-*hOGG1 *has a nuclear localization signal while β-*hOGG1 *is targeted to mitochondria [22,23]. *hOGG1 *has been shown to have both DNA glycosylase/AP lyase activity that preferentially removes 8-oxoG opposite cytosine. Inactivation of Fpg or MutY genes in *E. coli *or *OGG1 *in yeast leads to a spontaneous mutator phenotype characterized by the exclusive increase in G : C to T : A transversions [15,24-27]. Additionally, *OGG1*-deficient strains of *S. cerevisiae *have an increased frequency of mitochondrial mutants [28]. Other studies have shown that cellular survival and mtDNA repair can be enhanced by targeting wild type *hOGG1 *to the mitochondria [29,30], suggesting that this gene is critical for the maintenance of mitochondria genome and cellular survival in response to oxidative DNA damage. Accordingly, mutations in *hOGG1 *may affect mtDNA integrity and the ability of cells to survive under oxidative stress. *hOGG1 *mutations have been detected in human cancers [31,32]. In this study, we examined the effect of mutant *hogg1 *(R229Q) found in a leukemia cell line [33], on mtDNA integrity and cellular survival.

Current assays of DNA repair measure global DNA damage utilizing large quantities of DNA or involve whole cells [34,35]. DNA damage and repair at the gene level, has been performed using gene specific repair assay by alkaline gel electrophoresis and Southern hybridization which also require large quantities of DNA and radio-labeled ^32^P [36]. Quantitative PCR has been used to measure DNA repair at an individual gene level [37-39]. We used quantitative real time PCR to analyze mtDNA integrity after exposure to oxidative damaging agents, H_2_O_2 _and Adriamycin in cells transfected with or without mitochondrially-targeted mutant *hogg1*. We found that targeting R229Q mutant *hogg1 *to the mitochondria significantly reduced mtDNA integrity and resulted in decreased cellular survival after exposure to oxidative agents when compared to the wild type *hOGG1*. Our results also showed that mitochondrially targeted mutant *hogg1 *was more detrimental to cellular survival than nuclear targeted mutant *hogg1*.

## Methods

### Plasmid construction

Plasmid pCMV/myc/mito and pCMV/myc/nuc were obtained from Invitrogen, CA. *hOGG1 *was amplified using c-DNA from normal retinal epithelial cells (ARPE-19) using forward primer, 5' ACGGTCGACATGCCTGCCCGCGCGCTTCT 3' and reverse primer 5' AAGGAAAAAAGCGGCCGCGCCTTCCGGCCCTTTGGAAC 3' (underlined are *Sal I *and *Not I *sites) and cloned into to *Sal I*/*Not I *sites of plasmid pCMV/myc/mito resulting in pCMV/myc/mito-*hOGG1*. The cloned gene was then sequenced to rule out any mutation. Plasmid pCMV/myc/mito-*hOGG1 *(MTS-*hOGG1) *was then used to generate a mutant at amino acid position 229, by changing CGA (Arginine) to CAA (Glutamine) using primers 5' CTGGCTGCAGCAGCTACAAGAGTCCTCATATGAG 3' and its reverse complement, using the site directed mutagenesis kit (Stratagene, La Jolla, CA). The generated mutant plasmid was called pCMV/myc/mito-mutant-*hogg1 *(MTS-mutant-*hogg1*). Again the generated plasmid was sequenced to rule out any mutation other than the desired point mutation at codon 229. pCMV/myc/mito-*hOGG1 *and pCMV/myc/mito-mutant-*hogg1 *were digested with *Sal I *and *Not I*. Both the wild type and mutant *hOGG1 *were cloned into to *Sal I */*Not I *sites of plasmid pCMV/myc/nuc resulting in plasmids pCMV/myc/nuc-*hOGG1 *(Nuc-*hOGG1*) and pCMV/myc/nuc-mutant-*hogg1 *(Nuc-mutant-*hogg1*) respectively. MTS represents mitochondrial targeted sequence and Nuc represents nuclear targeted sequence. The generated plasmids were sequenced to rule out any mutations.

### Cell culture

HeLa cells were transfected with plasmid MTS-*hOGG1*, MTS-mutant-*hogg1*, Nuc-*hOGG1*, Nuc-mutant-*hogg1*, and empty vector (Invitrogen, Carlsbad CA) using Fugene 6 (Roche, Indianapolis IN) in the ratio of 1:6 (DNA in μg: Fugene in μl). Transfected HeLa cells were maintained in DMEM low glucose supplemented with 10% FBS (Hyclone, Logan, UT) and 5% Penicillin-Streptomycin. All experiments were performed at 72 h post transfection, for maximum transfection efficiency.

### Preparation of mitochondrial and nuclear fractions

One T75 flask of each cell type (empty vector, MTS-*hOGG1*, MTS-mutant-*hogg1*, Nuc-*hOGG1 *and Nuc-mutant-*hogg1 *– transfected HeLa cells) were harvested at 72 h post transfection and washed once with ice cold 1X PBS. The cells were then treated with 0.04% ice-cold digitonin solution {0.4 mg Digitonin/ml; 2.5 mM EDTA, 250 mM Mannitol; 17 mM MOPS (pH 7.4)}, re-suspended and the contents dounce-homogenized with 10 strokes. To the homogenized cells, sucrose-mannitol buffer {525 mM Mannitol; 175 mM Sucrose; 12.5 mM Tris-HCl (pH 7.4)} was added and further dounce homogenized with 20 strokes. A small aliquot (20 μl) was observed under the microscope to assure complete disruption of cells. The cells were centrifuged at 2500 rpm in a microfuge for 10 min at 4°C. The resulting pellet was saved for nuclear protein extraction and the supernatant was re-centrifuged at 2500 rpm for 10 min at 4°C until no further pellet was visible. There after the supernatant was centrifuged at 14000 rpm (Eppendorff microfuge) for 20 min at 4°C. The obtained mitochondrial pellet was re-suspended in 1X sucrose-mannitol buffer and centrifuged at 14000 rpm for 20 min at 4°C. Proteins were isolated from the mitochondrial and nuclear pellets using RIPA buffer containing proteases inhibitors. The protein concentration was determined using the Bio-Rad protein estimation kit (Bio-Rad, Hercules, CA) as per manufacturer's recommendation.

### Total cellular extract

One T75 flask of empty vector transfected HeLa cells was harvested at 72 h post transfection and washed once with ice cold 1X PBS. The cells were then suspended in RIPA buffer (10 mM Tris pH 7.4, 150 mM NaCl, 5 mM EDTA, 1% Triton-X-100, 0.1% SDS) containing protease inhibitors (Roche, cat 1697498) and 1 mM PMSF (added fresh at all times), sonicated for 1 min. The cells were then centrifuged at 4°C at 14000 rpm in a microfuge, and the supernatant (cellular lysate) was stored at -80°C for further experiments.

### Western Blot analysis

Twenty microgram of protein lysates were separated on standard SDS-Polyacrylamide gel electrophoresis. The proteins were transferred on to a PVDF membrane at 200 mA for 1h. The membrane was blocked with 5% non-fat dry milk and phosphate buffered saline (PBS) with 0.1% Tween-20 (PBS-T) at room temperature for 1h, and then treated with polyclonal anti-*hOGG1 *antibodies (Novus Biologicals, Littleton, CO) in the presence of 5% non-fat dry milk and PBS-T over night at 4°C. All washings were done with PBS-T. The membrane was washed 4 times, 5 minutes each and treated with anti- rabbit HRP conjugate in the presence of 5% milk with PBS-T for 1 h at room temperature. The membrane was washed again 8 times, 5 minutes each and developed with Amersham developer as per manufacturer's instructions. Loading controls for mitochondrial extracts were performed by using cytochrome c oxidase II (*Cox II*)antibody (obtained from Molecular Probes, Eugene, OR) and Lamin B (Santa Cruz, CA) was used for nuclear extracts.

### Cell viability assay

HeLa-MTS-*hOGG1*, HeLa-MTS-mutant-*hogg1*, HeLa-Nuc-*hOGG1*, HeLa-Nuc-mutant-*hogg1 *and HeLa-Vector were grown in 35 mm, 6-well culture plates for 72 h post transfection as this time point showed maximum transfection efficiency. The cells were rinsed with Hanks' Balanced Salt Solution (HBSS) and treated with 0, 100, 200, 400, 500 and 600 μM of H_2_O_2 _for 2 h. The MTS-*hOGG1 *and vector only transfected cells were also treated with 0, 20, 40, 60, 80, 100 μM of 4-nitroquinoline 1-oxide (4NQO) for 1 h in serum free media at 37°C in 5% CO_2 _incubator. After the desired time of exposure, the drug containing medium was aspirated, the cells were rinsed with HBSS and then allowed to recover in 1 ml of regular growth medium for 16 h. 100 μl MTT (3-(4,5-dimethylthiazol-2yl)-2,5 diphenyltetrazolium bromide) solution was added to each well and incubated at 37°C and 5% CO_2_. Four hours following incubation, 1 ml solubilization buffer was added and the mixture was incubated overnight at 37°C to allow complete solubilization. Spectrophotometric readings (A_570 nm _– A_650 nm_) were obtained on a Molecular Devices Spectra Max 250, 96 well plate reader (Sunnyvale, CA). The percent survival was calculated by assigning the (A_570 nm _– A_650 nm_) of the untreated cells to 100%. The ATCC – MTT Cell Proliferation Assay kit was used for all experiments.

### Drug preparation and exposure

HeLa-MTS-*hOGG1*, HeLa-MTS-mutant-*hogg1 *and HeLa-Vector were grown in 35 mm dishes for 72 h post transfection. The cells were rinsed with HBSS and then treated with 400 μM H_2_O_2 _and 28 μM Adriamycin for 2 h each and 50 μM 4NQO for 1 h in serum free medium at 37°C in 5% CO_2 _incubator. After the desired exposure time, the drug containing medium was removed, the cells were rinsed again with HBSS. To isolate the DNA, the cells were trypsinized, washed with PBS, and incubated in lysis buffer {100 mM NaCl; 10 mM Tris-HCl (pH 8); 0.25 mM EDTA; 0.5% SDS} containing 100 μg/ml proteinase K for 24 h at 48°C. DNA was extracted by a standard phenol-chloroform procedure followed by alcohol precipitation.

### Quantitative Real-Time PCR

The 7900HT sequence-detection system (Applied Biosystems) was used to perform real-time PCR amplification for nuclear β-actin and the mtDNA regions cytochrome *c *oxidase (*Cox I *and *Cox II*), D-loop1 (401–490), and D310. Table 1 lists the primers and probes used to amplify the respective DNA regions. All primers were obtained from Invitrogen (Carlsbad, CA). All TaqMan probes (Applied Biosystems, Foster City, CA) were labeled with 5'-FAM (6-carboxyfluorescein, fluorescent reporter) and 3'-TAMRA (6-carboxy-tetramethylrhodamine, fluorescence quencher). PCR amplifications were carried out in buffer containing 16.6 mM ammonium sulfate, 67 mM Tris base, 2.5 mM MgCl_2_, 10 mM 2-mercaptoethanol, 0.1% DMSO, 0.2 mM each of dATP, dCTP, dGTP, and dTTP, 600 nM each of forward and reverse primers, 200 nM TaqMan probe, 0.6unit Platinum *Taq *polymerase, and 2% Rox reference dye. DNA (1 ng) was used to amplify both the mitochondrial regions and the β-actin. The real-time PCR reactions were performed in triplicate for each gene and standard curves were obtained by using HeLa DNA from untreated cells. Data analysis was performed by using Microsoft EXCEL software. mtDNA/nDNA ratios were calculated by dividing the mtDNA signal for each gene by the β-actin signal and expressing the ratio as a percentage of the untreated control set at 100%.

## Results

### Over expression of mutant *hogg1 *in mitochondria

**Figure 1:**
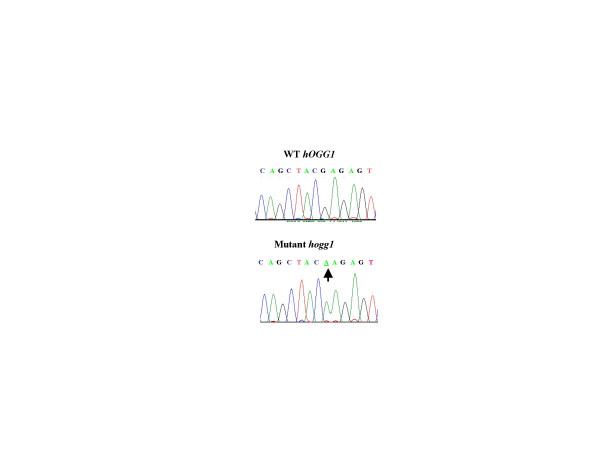
**Sequence of *hogg1 *Mutant**. *hOGG1 *(wild type) and mutant-*hogg1 *(mutant) were transfected in HeLa cells. 72 h post transfection, total RNA was isolated and RT-PCR was performed using *Superscript II *(Invitrogen, Carlsbad, CA) as per manufacturer's instructions. Automated DNA Sequencing results indicated that cells transfected with mutant-*hogg1 *harbored mutant *hogg1*, where CGA is changed to CAA at codon 229. Arrow indicates mutated position (G to A).

**Figure 2:**
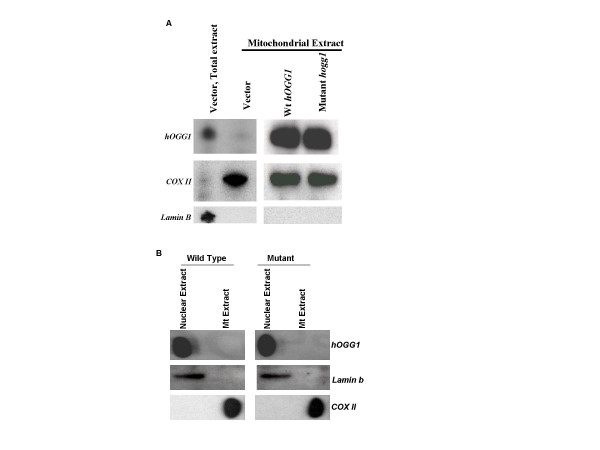
**Expression of *hOGG1 *targeted to mitochondria**. HeLa cells were transfected with empty vector, MTS-*hOGG1*, MTS-mutant-*hogg1*, Nuc-*hOGG1*, and Nuc-mutant-*hogg1*. Mitochondrial, nuclear and total cellular extracts were isolated and analyzed by Western blot analysis using anti-*OGG1 *antiserum. Twenty microgram of mitochondrial, nuclear and total cellular extract for each indicated transfection was loaded into each lane (Figure 2A. & B.). Protein extracts in each lane are as indicated. Immunodetection of *Lamin B *and *COX II *was done to assure that the transfected proteins were in nucleus and mitochondria respectively.

We transfected HeLa cells with mitochondrially-targeted wild type *hOGG1*, mutant *hogg1*, or the empty vector. Figure 1 shows that at 72 h post transfection DNA from the cells transfected with mutant-*hogg1 *actually harbored the mutation Arg229Gln. To show that *hOGG1 *protein was specifically expressed in the mitochondria, we performed western blot analysis on total cell extract and mitochondrial extracts after transfection. As observed in vector transfected HeLa cells, *hOGG1 *was expressed in the total cell extract, but virtually absent in the mitochondrial fraction (Figure 2A, Vector- total extract and Vector- mitochondrial extract), confirming that HeLa cells lack *hOGG1 *protein in their mitochondria. Furthermore, when compared to the vector only transfected cells, mitochondrial extracts of the wild-type MTS-*hOGG1 *and the MTS-mutant-*hogg1 *showed a clear over expression (more than 100 fold) of the 39 kD *hOGG1 *protein (Figure 2A, Wt *hOGG1 *and mutant *hogg1*). The R229Q mutation did not affect the expression and transportation of *hOGG1 *as there was no difference in the mitochondrial expression when compared to the wild type *hOGG1*. The nuclear targeted wild-type *hOGG1 *and the mutant-*hogg1 *showed a robust over expression in the nucleus only (Compare vector, total extract in Figure 2A, with nuclear extract in Figure 2B). Targeting of *hOGG1 *to the nucleus did not result in translocation to the mitochondria (Figure 2B, Mt extract). In order to rule out contamination from non-mitochondria or non-nuclear fractions, the membranes were washed and hybridized with mitochondrial *Cox II *and nuclear envelope Lamin B antibody.

### Effect of mutant *hogg1 *on cell viability

**Figure 3:**
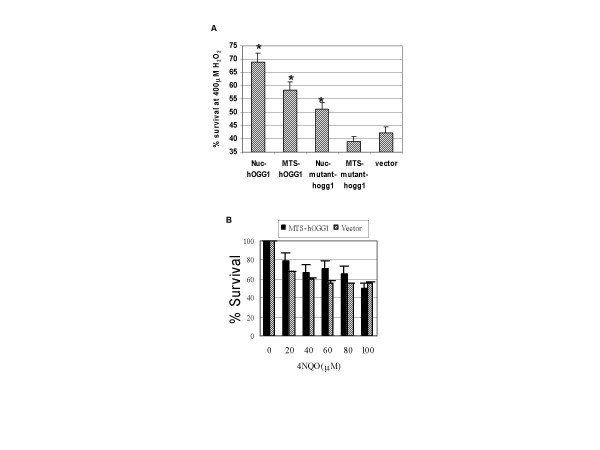
**Cell Survival of *hOGG1 *Regulated Cells**. HeLa cells transfected with indicated plasmids were grown in regular growth media, for 72 h post transfection. The cells were plated at a density such that they reached a 70% confluence on the day of the treatment. The cells were then treated with 400 μM H_2_O_2 _(A) for 2 h and for 1 h with 4NQO (B) in serum free media, and allowed to recover in normal growth medium for 16 h. Cellular survival was assessed using the MTT cell proliferation assay kit. Error bars represent standard deviation of four points. Nuc-*hOGG1 *(nuclear targeted *hOGG1*); MTS-*hOGG1 *(mitochondrially-targeted *hOGG1*), Nuc-mutant-*hogg1 *(nuclear targeted mutant *hogg1*), MTS-mutant-*hogg1 *(mitochondrially-targeted mutant *hogg1*), Vector (empty vector). *p *< 0.05 when the data from Nuc-*hOGG1*, MTS-*hOGG1 *and Nuc-Mutant-*hogg1 *transfectants was compared with vector-only cells using Student's *t *test.

Mitochondrial DNA damage may alter mitochondrial function, consequently affecting cell growth. To determine whether over expression of mutant *hogg1 *in mitochondria and nucleus had any effect on cellular survival following oxidative stress, we performed the MTT cell proliferation assay. MTT is a tetrazolium salt that is reduced by fully functioning mitochondria and results in a change of color from yellow to purple. Thus, the reduction of MTT can be monitored spectrophotometrically [40]. Therefore, a change in mitochondrial function and cell viability can be assayed using MTT as previously shown [39]. HeLa cells transfected with MTS-mutant *hogg1 *(Arg229Gln) were more sensitive to oxidative damage when compared to cells transfected with wild type MTS-*hOGG1 *(Figure 3A). Our results also showed that mitochondrially-targeted mutant *hogg1 *resulted in decreased cell survival compared to nuclear targeted mutant *hogg1 *upon oxidative damage with 400 μM H_2_O_2 _(Figure 3A, compare Nuc-mutant-*hogg1 *and MTS-mutant-*hogg1*). Statistical analysis using student's t-test revealed that p value for Nuc-*hOGG1*, MTS-*hOGG1 *and Nuc-mutant-*hogg1 *was 0.0004; 0.008 and 0.029 respectively. When the wild type *MTS-hOGG1 *and vector only transfectants were treated with varying concentrations of 4NQO, there was a modest decrease in cellular survival however, there was no significant difference between MTS-*hOGG1 *and vector transfected cells, (Figure 3B), again confirming that *hOGG1 *has little, if any effect on 4NQO-induced damage.

### Mutant *hogg1 *and mtDNA integrity

We used quantitative real time PCR to analyze mtDNA integrity after exposure to oxidative damaging agents, in the presence and absence of mitochondrially-targeted wild type *hOGG1 *or mutant *hogg1*. Quantitative real-time PCR assay allows for the measurement of DNA damage in any individual amplifiable DNA segment. The fundamental principle of this assay is that DNA damage will impede the progression of the DNA polymerase used in the PCR reactions [38,39]. Thus, DNA damage is detected as a reduction of the available template for PCR (decreased DNA integrity), resulting in a shift of the amplification curve to the right [39]. The major advantage of the quantitative real-time PCR assay is that only nanogram quantities of DNA are required, and DNA damage can be assessed at the individual gene level. Further this method enables the monitoring of mtDNA integrity directly from total cellular DNA without the need for isolating mitochondria, or a separate step of mtDNA purification [41].

**Figure 4:**
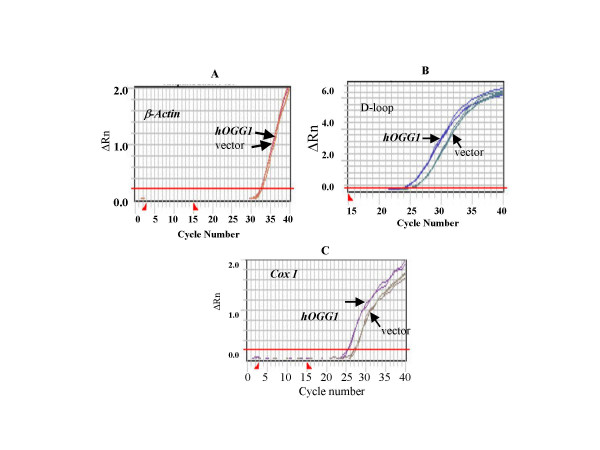
**Real Time PCR Amplification Curves**. Representative real-time PCR amplification curves generated for nuclear β-actin gene (Fig. 4A.), mitochondrial D-Loop (Fig. 4B.) and *Cox I *(Fig. 4C.) with and without wild type MTS-*hOGG1 *after treatment with 400 μM H_2_O_2_. Each experiment was performed in triplicate and is shown by overlapping amplification curves. ΔRn = (Rn^+^) - (Rn^-^), where Rn^+ ^is the fluorescence emission intensity of reporter/emission intensity of quencher at any time point, and Rn^- ^is the initial emission intensity of reporter/emission intensity of quencher in the same reaction vessel before PCR amplification is initiated.

We performed quantitative real-time PCR amplification for the nuclear β-actin gene and specific mtDNA regions: cytochrome *c *oxidase (*Cox I *and *Cox II*), D-loop (401–490), and D310 [37]. The extent of decrease in mtDNA integrity was analyzed by calculating the mtDNA/nuclear DNA (β-actin) ratio, and normalizing to the untreated control set at 100%. Ratios of mtDNA/nDNA were used to obtain the relative DNA integrity whereby a lower ratio represents less initial template, denoting a decrease in the integrity of mtDNA. The mtDNA/nDNA ratio of the untreated HeLa-Vector was set at 100%. Similarly, the mtDNA/nDNA ratio of the untreated *MTS-hOGG1 *and *MTS-*mutant-*hogg1 *were set at 100% for calculating values obtained with recombinant wild-type (*hOGG1*) or mutant (*hogg1*) respectively. Figure 4 shows representative real-time PCR amplification curves of β-actin (Figure 4A), D-Loop (Figure 4B) and *Cox I *(Figure 4C) using DNA isolated from HeLa-Vector and HeLa-MTS-*hOGG1 *(wt) transfected cells treated with 400 μM H_2_O_2 _for 2 h. At the concentration of H_2_O_2 _used in these studies, there was no damage to the β-actin region as evidenced by the overlapping curves in the absence (vector) and presence of wild type *hOGG1 *(Figure 4A). However amplification curves of both D-loop and *Cox I *shifted to the right in the absence (vector) of wild type *hOGG1*, showing a one cycle difference (2 fold decrease in amplifiable template). This indicates that recombinant *hOGG1 *is efficient in maintaining the mtDNA integrity after oxidative damage.

**Figure 5:**
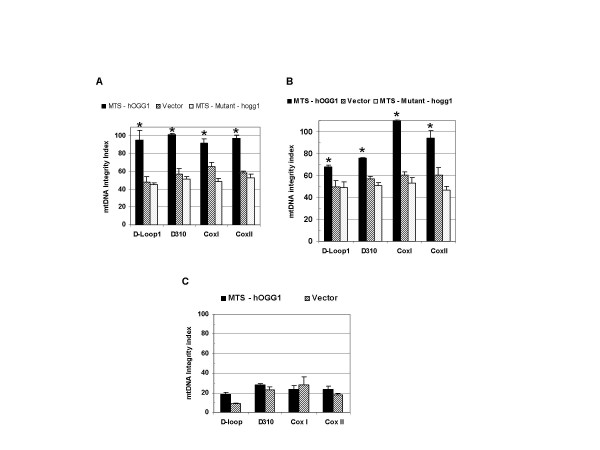
**mtDNA integrity after oxidative damage exposure**. MtDNA integrity of indicated mitochondrial regions in HeLa cells transfected with empty vector, MTS-*hOGG1 *and MTS-Mutant *hogg1 *and treated with (A) 400 μM of H_2_O_2 _for 2 h; (B) 28 μM of Adriamycin for 2 h and (C) 50 μM of 4-NQO for 1 h were analyzed by using quantitative real-time PCR amplification. The extent of decrease in mtDNA integrity was analyzed by calculating the mtDNA/nuclear DNA ratio, and normalizing to the untreated control set at 100%. The error bars represent standard deviation of each experiment done twice in triplicates. An *asterisk *indicates a significant difference (0.004 ≥ *p *≥ 0.0001), when compared to the vector using Student's *t *test.

A quantitative analysis of the damage induced by H_2_O_2 _to the different mitochondrial regions is depicted in Figure 5A. When compared to the wild type *hOGG1 *(black bars), both the vector only (hatched bars) and mutant *hogg1 *(spotted bars) transfected cells had approximately 2 fold reduction in mtDNA integrity as evidenced by the reduced ratios. Little to no damage was observed in all regions in the presence of wild type MTS-*hOGG1*, showing that over-expression of wild type *hOGG1 *limited damage to the mtDNA during the 2 h concurrent damage/repair time. On the contrary, over-expression of a mutant *hogg1 *was abortive in protecting against oxidative damage to mtDNA in all regions analyzed (Figure 5A). Statistical analysis using student's t-test revealed that p value for D-loop1, D310, *COX I* and *COX II* was 0.0001; 0.004; 0.004 and 0.003 respectively. A similar trend was observed with Adriamycin (Figure 5B). Over-expression of mutant *hogg1 *resulted in reduced mtDNA integrity compared to wild type *hOGG1 *(MTS-*hOGG1*) after exposure to Adriamycin, indicating that the R229Q *hOGG1 *mutation compromised mtDNA integrity. Conversely, over-expression of the wild type *hOGG1 *resulted in no damage to the transcribed regions (*Cox I *and *Cox II*), and less damage to the control region (D-loop and D-loop1) (Figure 5B), indicating that over expression and localization of *hOGG1 *to mitochondria enhanced mtDNA integrity. A statistical analysis for figure 5B using student's t-test revealed that p value for D-loop1, D310, *COX I* and *COX II* was 0.0005; 0.0006; 0.0001 and 0.001 respectively. Our results clearly indicate that the Arg229Gln amino acid change was unable to protect mtDNA integrity from oxidative damage(s). Interestingly, when the cells were exposed to 4NQO, both the wild type (*hOGG1*) and the vector transfected cells were significantly damaged (as evident from the low ratios) resulting in a significant reduction in mtDNA integrity (Figure 5C). These observations indicate that the major lesions induced by 4NQO are not repaired by *hOGG1*. 4NQO is known to induce lesions that are mainly repaired through the nucleotide excision repair (NER) pathway, exclusive of *hOGG1*.

## Discussion

Understanding the maintenance of mtDNA integrity and its contribution to normal cellular survival is vital to unraveling human mitochondrial diseases. mtDNA mutations have been found in patients with a variety of chronic diseases and cancer [1,2,42,43]. Moreover, increase in 8-oxoG levels as well as rare *hOGG1 *mutations were reported in various types of human cancer [1,2,32,42-45]. It It has been speculated that damage to mtDNA may be important in determining cellular survival and that lack of repair of mtDNA could result in initiating the mitochondrial-dependent apoptotic pathway and increased cell death. Mutations in *hOGG1 *may affect mtDNA integrity, and the ability of cells to survive under oxidative stress. In this study, we examined the effects of a human leukemia R229Q mutation in the DNA repair gene *hOGG1 *on mtDNA integrity and cellular survival.

We used HeLa cells to examine the effect of mitochondrially and nuclear-targeted mutant *hogg1 *on cellular survival and mtDNA integrity. HeLa cells have normal protein expression of nuclear *hOGG1*, but lack expression of *hOGG1 *protein in the mitochondria [30]. Thus, HeLa cells provide an excellent model for studying the effects of mitochondrially-targeted *hOGG1*. When compared to the wild type *hOGG1*, our Western blot results showed that the R229Q mutation did not affect the expression level of *hogg1 *protein. Furthermore, our western blot also suggested that the nuclear-targeted mutant *hogg1 *was confined to the nucleus only. Our results showed that targeting and over-expression of the R229Q mutant *hogg1 *to the mitochondria resulted in a reduction of both cellular survival and mtDNA integrity after oxidative damage. Over-expression of mutant *hogg1 *in both nucleus and mitochondria also failed to protect the cells from oxidative damage when compared to over-expression of the wild type *hOGG1*. However, mitochondrially-targeted mutant *hogg1 *was more detrimental to cellular survival than nuclear-targeted mutant *hogg1 *upon oxidative damage. Previous results by Hyun *et al *[33] showed that R229Q mutation resulted in decreased *hOGG1 *enzymatic activity as measured by *in vitro *8-oxoG incision assay. Together these results indicate that functional *hOGG1 *is critical and required for maintenance of mitochondrial genome and cellular response to oxidative damage. Additionally, we show that over-expression of wild type *hOGG1 *in the mitochondria resulted in increased mtDNA integrity in both the control and coding regions, and enhanced cellular survival after oxidative damage exposure. H_2_O_2 _has been shown to induce a wide variety of lesions, including strand breaks and at least 11 major different base oxidations [46]. Among these, 8-oxoG is the most stable and has long been suspected to play an important role in the initiation of carcinogenesis [47-51]. Recently we have shown that with decreased expression of *hOGG1 *in lung cell lines, there is an increase in 8-oxoG levels coupled with a decrease in mtDNA integrity due to increased damage of mtDNA, upon exposure to H_2_O_2 _treatment [52]. Further, the generation of mice deficient in the repair 8-oxoG (*ogg1*-/- mice) has opened the door for alternative approaches. Results obtained from *hOGG1 *knockout animals indicated an increase in 8-oxoG lesions in the liver, and a higher spontaneous mutation frequency [53]. Other studies have shown that *hOGG1 *knockout mice developed lung tumors spontaneously with increased 8-oxoG in their DNA [54]. However, there is still limited information on *hOGG1 *and integrity of mtDNA.

Previous results [30] showed that over-expression of wild type *hOGG1 *enhanced mtDNA repair and cellular survival. In this report, we showed that the R229Q mutant *hogg1 *caused a decrease in mtDNA integrity and sensitized cells to induced oxidative damage. Together our mutant *hogg1 *results and those of Rachek *et al*., [30] highlight the importance of fully functional *hOGG1 *in cellular protection against ROS, and further that this gene is required for efficient maintenance of mtDNA integrity and cellular survival. Additionally, our results show that oxidative damage to mtDNA may contribute to cellular sensitivity suggesting that mtDNA is a key determinant and that its excessive damage may trigger cell death pathways.

We also treated cells with 4NQO, a UV-mimetic agent that induces a wide range of lesions including DNA adducts, single-strand breaks, pyrimidine dimmers, abasic sites, and perhaps a limited amount of oxidized bases [55]. These lesions are mainly repaired through nucleotide excision-repair, a mechanism not yet established in the mitochondria. Although the cells were moderately sensitive to 4NQO, we found no significant difference in the survival pattern of the *MTS-hOGG1 *or the vector only transfected cells following exposure to 4NQO. Furthermore, the decrease in mtDNA integrity by 4NQO was not affected by the presence of *MTS-hOGG1*, suggesting that 4NQO did not induce damage that is repaired by *hOGG1*. We attribute this result to the narrow substrate specificity of *hOGG1 *which specifically repairs 8-oxoG opposite cytosine [18,19,56,57] and has little or no affinity for other lesions.

We used quantitative real-time PCR to analyze mtDNA integrity in cells with or without mitochondrially-targeted wild type *hOGG1 *and mutant *hogg1*. The assay is based on the principle that DNA damage will impede the progression of the DNA polymerases used in the PCR reactions [37-39,58]. Although some polymerases like yeast and human pol η can bypass 8-oxoG efficiently and accurately, other polymerases like yeast pol δ have been shown to stall at or just before the lesion, only by-passing about 14% of the time [59]. *E. coli *RNA polymerase and mammalian RNA polymerase II have also been shown to stall at 8-oxoG lesions, resulting in decreased transcript formation [60,61]. These findings indicate that 8-oxoG can impede both DNA and RNA polymerases, interfering with transcription and replication of DNA. Our quantitative PCR assay was able to assess and distinguish mtDNA integrity in MTS-*hOGG1*, MTS-mutant-*hogg1 *and vector only transfected cells after oxidative damage exposure. Our results showed clear differences in mtDNA integrity between wild type MTS-*hOGG1*, MTS-mutant-*hogg1 *and the vector only transfected cells. Because of the distinct differences observed between wild type and mutant *hOGG1*, our findings suggest that the Taq polymerase used did not efficiently bypass 8-oxoG lesion, rather it had limitations. However, the role of 8-oxoG in blocking polymerases still remains controversial. Recent reports show that the use of quantitative real time PCR (QPCR) is very useful in measuring the integrity of both nuclear and mitochondrial genomes exposed to different genotoxins, and has proved particularly valuable in identifying reactive oxygen species-mediated mitochondrial DNA (mtDNA) damage. [41].

The results from the present study showed that mitochondrially-targeted *hOGG1 *plays a crucial role in maintaining mtDNA integrity and cellular survival.

## Conclusion

Our results demonstrate that functionally compromised *hogg1 *mutants in the mitochondria compromised mtDNA integrity. Furthermore, the presence of mutant *hogg1 *in the mitochondria failed to protect cells from oxidative damage, more than when the mutant *hogg1 *was present in the nucleus. *hOGG1 *alterations and point mutations occur in human cancers, suggesting that aberrant *hOGG1 *function may increase both nuclear and mtDNA mutation loads. It is also believed that *hOGG1 *polymorphic variants may predispose individuals to cancer. Thus targeting other *hOGG1 *variants or mutants to the mitochondria will help us further elucidate their role in cancer and other human diseases.

## Abbreviations

mtDNA, mitochondrial DNA; nDNA, nuclear DNA; Cox I, cytochrome c oxidase subunit I; 4-NQO, 4-nitroquinoline 1-oxide; H_2_O_2_, hydrogen peroxide; *hOGG1*, human 8-oxoguanine DNA glycosylase; 8-oxoG, 7,8-dihydro-8-oxoguanine; BER, base excision repair; ROS, reactive oxygen species; MTS, mitochondrial targeting sequence; AP, apurinic/apyrimidinic; FBS, fetal bovine serum; DMEM, Dulbecco's modified eagles medium; MOPs, 4-morpholinepropanesulfonic acid; PBS, phosphate buffered saline; PBS-T, phosphate buffered saline – tween 20; SDS, sodium dodecyle sulfate; EDTA, Ethylenediaminetetraacetic acid; MTT, (3-(4,5-diméthylthiazol-2yl)-2,5 diphényltétrazolium bromide).

## Competing interests

The author(s) declare that they have no competing interests.

## Authors' contributions

AC did plasmid preparation, cell culture, preparation of mitochondrial and nuclear fractions and western blot analysis and Real time PCR. EM did drug treatment, cell viability, Real time PCR and western blot analysis. Both AC and EM have been involved in acquisition, analysis and interpretation of data, and in drafting the manuscript. YZ revised the manuscript critically for important intellectual content. TD and DS are the senior authors who have given final approval of the version to be published.

## Pre-publication history

The pre-publication history for this paper can be accessed here:



## Figures and Tables

**Table 1:** Sequence of Primers and Probes used in the Quantitative Real-time PCR Analysis.

**Region**	**Forward primer (5'-3')**	**Reverse primer (5'-3')**	**TaqMan probe (5'-3')**
**D-Loop**	tatcttttggcggtatgcacttttaacagt (401–430)	tgatgagattagtagtatggg (487–467)	caccccccaactaacacattattttcccc (431–459)
**D310**	cacacagacatcataacaaaaaatttcc (269–296)	ggtgttagggttctttgtttttgg (378–355)	ccccccctcccccgcttct (303–321)
**Cox II**	ccccacattaggcttaaaaacagat (8080–8104)	tatacccccggtcgtgtagc (8160–8141)	caattcccggacgtctaaaccaaaccactttc (8106–8137)
**Cox I**	ttcgccgaccgttgactattctct (6007–6030)	aagattattacaaatgcatgggc (6103–6081)	aacgaccacatctacaacgttatcgtcac (6051–6079)
**β-Actin**	tcacccacactgtgcccatctacga (2141–2165)	cagcggaaccgctcattgccaatgg (2435–2411)	atgccctcccccatgccatcctgcgt (2171–2196)
